# Glutamate is effective in decreasing opacity formed in galactose-induced cataract model

**DOI:** 10.1038/s41598-024-54559-y

**Published:** 2024-02-19

**Authors:** Masaru Takashima, Shunki Yamamura, Chie Tamiya, Mayumi Inami, Yoshihiro Takamura, Masaru Inatani, Masaya Oki

**Affiliations:** 1 Department of Industrial Creation Engineering, Graduate School of Engineering, University of Fukui, Fukui, Japan; 2 Technical Division, School of Engineering, University of Fukui, Fukui, Japan; 3 Department of Ophthalmology, Faculty of Medical Sciences, University of Fukui, Fukui, Japan; 4 Life Science Innovation Center, University of Fukui, Fukui, Japan

**Keywords:** Lens diseases, Transcription

## Abstract

Although cataract is the leading cause of blindness worldwide, the detailed pathogenesis of cataract remains unclear, and clinically useful drug treatments are still lacking. In this study, we examined the effects of glutamate using an ex vivo model in which rat lens is cultured in a galactose-containing medium to induce opacity formation. After inducing lens opacity formation in galactose medium, glutamate was added, and the opacity decreased when the culture was continued. Next, microarray analysis was performed using samples in which the opacity was reduced by glutamate, and genes whose expression increased with galactose culture and decreased with the addition of glutamate were extracted. Subsequently, STRING analysis was performed on a group of genes that showed variation as a result of quantitative measurement of gene expression by RT-qPCR. The results suggest that apoptosis, oxidative stress, endoplasmic reticulum (ER) stress, cell proliferation, epithelial-mesenchymal transition (EMT), cytoskeleton, and histones are involved in the formation and reduction of opacity. Therefore, glutamate may reduce opacity by inhibiting oxidative stress and its downstream functions, and by regulating the cytoskeleton and cell proliferation.

## Introduction

Cataract is characterized by lens opacity, vision loss, and blindness, and is the leading cause of blindness worldwide^1^. Currently, the sole treatment for cataracts is the surgical insertion of artificial lenses^2^, a procedure often unavailable in developing countries with less advanced medical systems. Therefore, pharmacological approach to treatment is desired.

Cataracts develop due to a variety of factors, including aging and exposure to ultraviolet light. However, diabetes accelerates cataract development and increases the risk by 2–5 times^3,4^. We prioritized diabetic cataracts due to the increasing number of diabetic patients year by year^5^. In diabetic patients, the prevalence of cortical opacities and posterior subcapsular opacities is high^6^. Known diabetic cataract models include the in vivo streptozotocin diabetes model^7^, galactose diet-loaded model^8^, and Nile grass rats^9^. Additionally, as an ex vivo model, a galactose-induced cataract model is known, in which the lens is cultured in a medium containing galactose^8^. Since we have previously screened various drugs in a galactose-induced cataract model, we used the same model in this study.

Mechanisms of diabetic cataract formation include the formation of membrane-impermeable sugar alcohols, non-enzymatic glycation, and oxidative stress^10^. In hyperglycemic conditions, aldose reductase (AR) produces membrane-impermeable sugar alcohols, increasing intracellular osmotic pressure and resulting in opacity^11^. Non-enzymatic glycation is the chronic production of methylglyoxal, an intermediate from sugar metabolism, in a high-sugar environment. Methylglyoxal binds to various proteins, causing loss of function and abnormal aggregation, ultimately resulting in opacity^12^. Furthermore, methylglyoxal may contribute to the development of diabetic complications, as it is also involved in the substrate-induced increase in AR^13^. Oxidative stress leads to opacity when accumulated reactive oxygen species (ROS), like hydrogen peroxide (H_2_O_2_), induce protein denaturation and cellular damage^14^. Furthermore, it has recently been reported that the induction of endoplasmic reticulum (ER) stress response, lens epithelial cell (LEC) apoptosis, and epithelial-mesenchymal transition (EMT) are implicated in the development of diabetic cataracts^15–17^. Therefore, a homeostatic breakdown of LECs may play a role in the development of diabetic cataracts.

Antioxidants are abundant in the lens and may prevent damage. However, hyperglycemia depletes antioxidants and weakens antioxidant mechanisms in the lens. For example, ROS is eliminated by reduced glutathione (GSH), which serves as a reducing agent. Oxidized glutathione (GSSG) is regenerated through a reduction reaction that utilizes NADPH as a coenzyme. On the other hand, AR also utilizes NADPH to convert glucose to sorbitol, which depletes intracellular NADPH, making GSSG irreducible and promoting oxidative stress^18^. This suggests that reducing oxidative stress with antioxidants is crucial for the prevention and treatment of cataracts.

To date, antioxidants have been demonstrated to prevent cataracts at an experimental level, but a fundamental treatment has not yet been realized. Therefore, in this study, we explored the effectiveness of antioxidants in the treatment of cataracts. Antioxidants that have been demonstrated to prevent cataracts at the experimental level include curcumin, vitamin C, vitamin E, and pyruvate ^19^. Among these, we concentrated on the pathway associated with pyruvate. Pyruvate has been demonstrated to prevent not only diabetic cataracts but also cataracts caused by selenite and ultraviolet radiation^20–23^. Pyruvate is an intermediate in the glycolytic metabolic pathway and is involved in metabolism within the TCA cycle and fatty acid metabolism. Pyruvate is converted to acetyl-CoA, which is subsequently metabolized in the TCA cycle. α-Ketoglutarate (α-KG), produced in the TCA cycle, is recognized for its ability to bind to and eliminate ROS^24,25^. Furthermore, glutamate can be converted to α-KG through the action of either glutamate dehydrogenase, alanine aminotransferase, or aspartate aminotransferase. Therefore, glutamate may possess antioxidant properties similar to those of pyruvate.

Glutamate is a non-essential amino acid that is also synthesized in the lens^26^. Glutamate is a component of GSH and may play a role in the formation of GSH^27^. Furthermore, the addition of proline, a metabolite derived from glutamate, to an H_2_O_2_ medium, followed by lens incubation, resulted in the prevention of opacity^28^. This suggests that glutamate may be effective in both the prevention and treatment of cataracts. However, the effect of glutamate on cataracts remains unclear, as subcutaneous administration of monosodium L-glutamate to newborn mice induces cataracts^29^.

In this study, we investigated whether the opacity formed in a galactose environment could be resolved by the addition of glutamate. Furthermore, genes whose expression was suppressed by the addition of glutamate were measured by RT-qPCR, and cataract-related genes were identified. These results indicate that glutamate may be effective as a therapeutic agent to reduce lens opacity in diabetic cataracts. We also suggest that further comparisons with agents that have shown therapeutic efficacy in the past in our laboratory may lead to novel approaches to elucidating the pathogenesis of diabetic cataracts^30,31^.

## Results

### Glutamate addition reduce opacity in galactose-induced cataracts in rats

**Figure 1:**
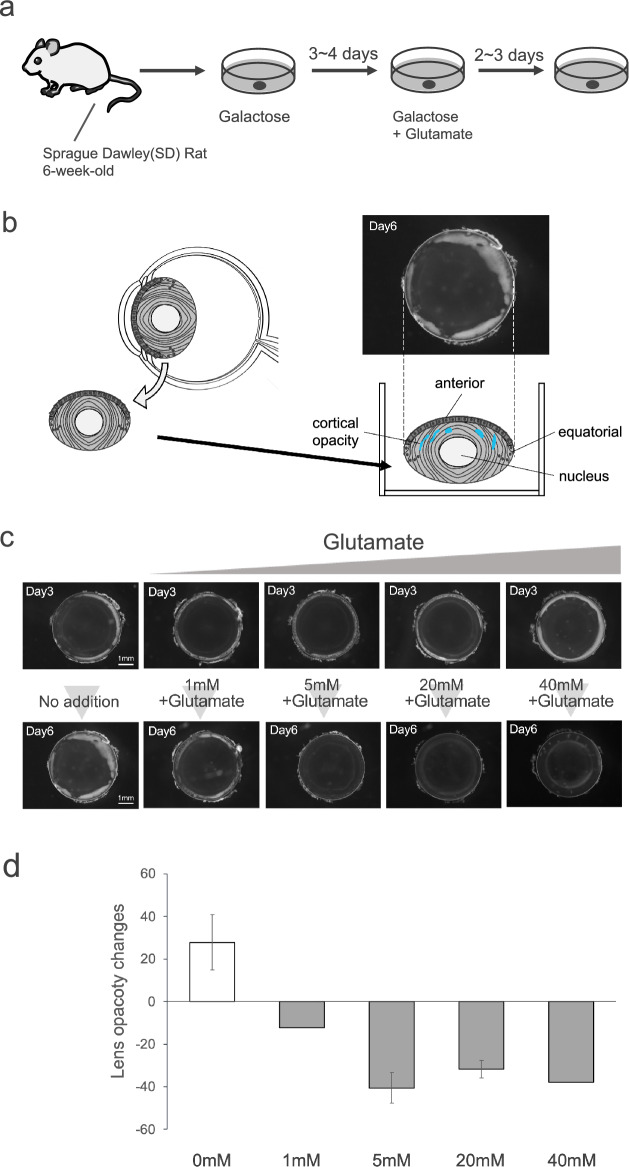
Effect of glutamate on lens opacity. (**a**) Scheme of the experiment using SD rat lens. (**b**) Position of the eyeball and lens. The lens photographs were taken from the anterior side, and each area is shown in the model diagram. (**c**) Rat lens cultured in 30 mM galactose-containing medium for 3–4 days (upper panel). In addition, after photographing the lens in the upper panel, sterile water as a vehicle control or glutamate dissolved in sterile water to final concentrations of 1, 5, 20, and 40 mM was added to the galactose-containing medium and cultured for 2–3 days. The number of days in the upper left panel indicates the total number of days of incubation. (**d**) The area of lens opacity without and with Glutamate was calculated, and the change in opacity before and after addition of the inhibitor was calculated. Data are expressed as mean ± SE. Samples used for quantification are shown in Supplementary Fig. S1.

In the past, pyruvate has been reported to prevent diabetic cataracts in animal studies^20,21^. Furthermore, α-KG, which is produced from pyruvate in the TCA cycle, is known for its ability to bind to and remove ROS, a contributor to oxidative stress^24^. Therefore, we examined whether glutamate, which produces α-KG as well as pyruvate, could reduce the opacity formed by galactose. Lenses were extracted from 6-week-old Sprague–Dawley (SD) rats and cultured in a medium containing galactose for 3–4 days to induce opacity in the equatorial cortex. The lenses were subsequently cultured for 2–3 days in a medium containing either galactose alone or a galactose medium with various concentrations of glutamate (1 mM, 5 mM, 20 mM, and 40 mM) to determine the glutamate concentration at which the opacity was reduced (Fig. 1a–d). Schematic diagram representing the portion of the eye in the photograph in Fig. 1c are shown in Fig. 1b. As a result, the opacity area of lenses cultured in a medium containing only galactose increased compared to the area before the medium change, whereas a reduction in opacity was observed in media with glutamate at all concentrations (Fig. 1c). Next, we measured the alteration in opacity before and after the addition of glutamate (Supplementary Fig. S1). The results demonstrated that the opacity decreased when glutamate was added at all concentrations (Fig. 1d). Since the minimum and maximum concentrations at which the effect of reducing opacity was observed were 5 mM and 40 mM, respectively, the analysis was performed at 20 mM, which is the concentration in the middle.

### Identification of genes involved in the decrease of opacity using microarrays

**Figure 2:**
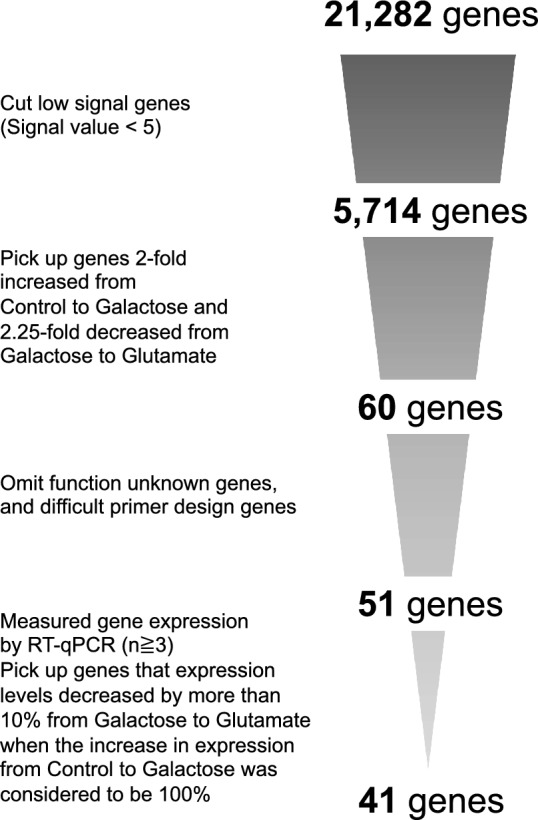
Flowchart for narrowing down the genes for which expression levels were altered upon Glutamate addition. 21,282 genes indicate the number of genes for which probes without gene names were deleted. 5714 genes indicate the number of genes after exclusion of genes with signal values of 5 or less in all samples. 60 genes indicate the number of genes whose expression levels increased more than two-fold from Control to Galactose and decreased more than two-fold from Galactose to Glutamate. 51 genes indicate the number of genes for which unknown functions or genes for which primer design was difficult were excluded. 41 genes are those for which the RT-qPCR results show that expression levels decreased by more than 10% from Galactose to Glutamate when the increase in expression from Control to Galactose was considered to be 100%, and the number of genes that matched the microarray analysis.

To identify the genes associated with the development of lens opacity induced by galactose and the effect of glutamate on opacity reduction, we cultured 2 samples (Control) for 4 and 6 days without galactose, 3 samples (Galactose) were cultured for 6 days in a medium containing galactose to induce opacity, and 2 samples (Glutamate) were cultured in a medium containing galactose for 3 days to induce opacity, followed by the addition of glutamate and an additional 3 days of culture for microarray analysis. A flowchart for the extraction of target genes is shown in Fig. 2. First, out of 36,685 probes, probes without gene names were deleted, and then genes with signal values of 5 or less in all samples were deleted from 21,282 genes, which reduced the number of genes to 5714. Subsequently, the average signal values among the Control, Galactose, and Glutamate samples were calculated, and the genes whose expression levels increased more than two-fold from the Control to Galactose and decreased more than 1.5-fold from Galactose to Glutamate were extracted. The number of genes whose expression levels increased more than two-fold from Control to Galactose and decreased more than 1.5-fold from Galactose to Glutamate was 265 genes (Supplementary Dataset S1). Furthermore, to narrow down the number of genes for RT-qPCR, 60 genes were extracted from the genes whose expression levels decreased by 2.25-fold or more from control to glutamate under the condition that the expression level increased by two-fold or more from control to galactose. In the subsequent experiments, we concentrated on the 60 genes that met the previously mentioned criteria and conducted an analysis.

**Figure 3:**
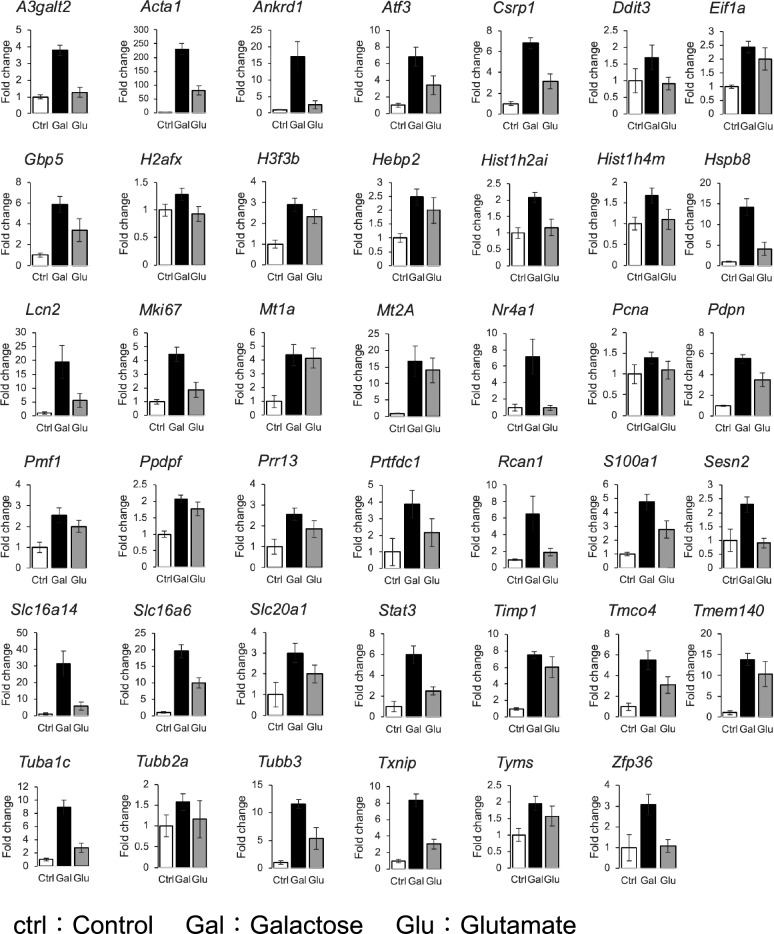
RT-qPCR results for genes with increased expression. RT-qPCR was conducted on 51 genes selected through microarray analysis (biological replicates/n ≧ 3). Out of these, 41 genes exhibited a decrease in expression levels by more than 10% from Galactose to Glutamate where the increase in expression from Control to Galactose considered as 100%. The results are presented as target gene mRNA levels normalized by *Gapdh* mRNA levels. Data are represented as the mean ± SE. Ctrl indicates Control; Gal, Galactose; and Glu, Glutamate.

To quantitatively measure the expression levels of the 60 genes extracted through microarray analysis, RT-qPCR was used. Before the analysis, genes for which primers could not be produced or for which function was unknown were excluded, leaving 51 genes to be measured by RT-qPCR. The details of these 51 genes are provided in Supplementary Table S1. In this study, the genes critical for reducing opacity were defined as those whose expression levels decreased by over 10% from Galactose to Glutamate, with the increase in gene expression from Control to Galactose considered as 100%. As in the microarray analysis, 41 of the 51 genes were increased from control to Galactose and decreased from Galactose to Glutamate (Fig. 3).

### Functional analysis of genes involved in decreasing opacity

**Figure 4:**
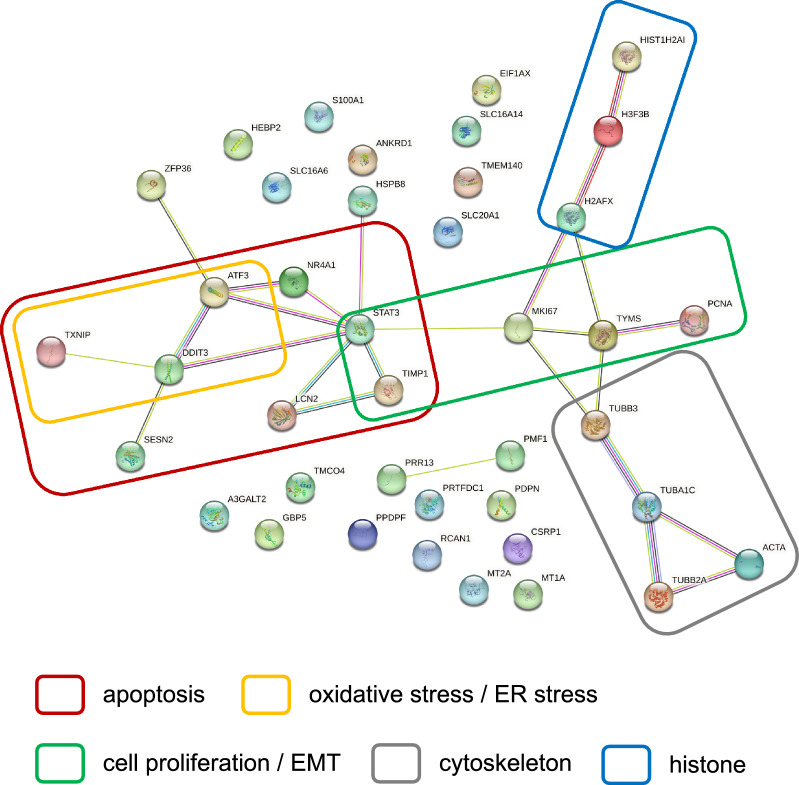
STRING protein interaction analysis. The results of STRING analysis of 41 genes with confirmed expression variation by RT-qPCR (https://string-db.org/). The selected organisms were *Homo sapiens*. Note that *Hist1h4m* was not converted to a human gene, and *Acta1* was converted to ACTA. The color of each edge indicates the type of relationship as follows: light blue = “from curated databases”; dark purple = “experimentally determined”; green = “text mining”; black = “co-expression”; and light purple = “protein homology”.

**Figure 5:**
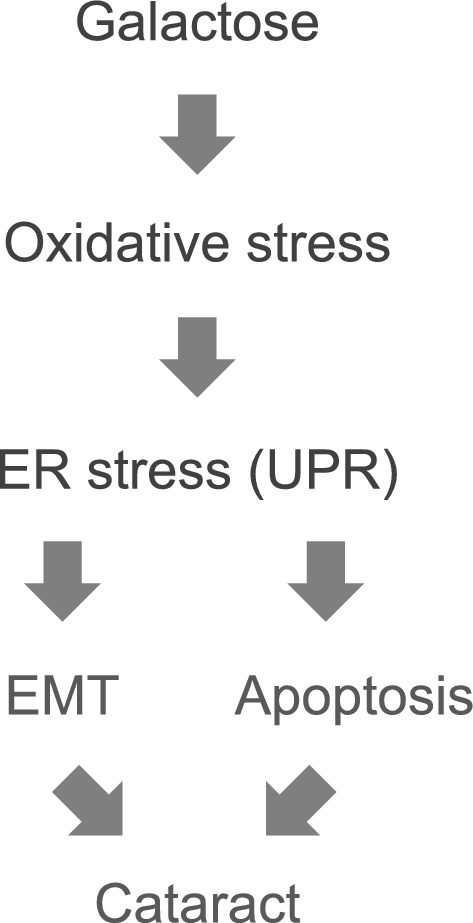
Predictive model of galactose-induced cataract. Oxidative stress-induced ER stress is triggered by the galactose environment. Subsequently, ER stress leads to apoptosis and EMT, ultimately culminating in cataract formation.

Subsequently, a protein–protein interaction analysis was conducted using STRING to elucidate the intracellular functions of the 41 genes (Fig. 4). Functional analysis revealed that 41 genes formed five groups: (1) apoptosis-related genes, (2) oxidative stress- and ER stress-related genes, (3) cell proliferation- and EMT-related genes, (4) cytoskeleton-related genes, and (5) histone-related genes. Apoptosis-related genes include *Atf3, Ddit3, Lcn2, Nr4a1, Sesn2, Stat3, Timp1, Txnip*^32–39^. Furthermore, among apoptosis-related genes, *Atf3, Ddit3,* and *Txnip* are involved in oxidative and ER stress^40–42^. *Atf3* is a stress-responsive transcription factor and *Ddit3* is a known apoptosis-promoting transcription factor^40,41^. *Txnip* is known to regulate intracellular redox as a thioredoxin-binding protein^43^. In fact, it has been reported that oxidative stress induces ER stress and the unfolded protein response (UPR) in lens epithelial cells, ultimately resulting in apoptosis^44^. Cell proliferation and EMT related genes include *Mki67, Pcna, Stat3, Timp1* and *Tyms*^45–49^. Among these, *Mki67* is known as a cell proliferation marker and *Pcna* as a proliferating cell nuclear antigen^45^. Furthermore, ER stress has been reported to induce EMT^50^. Next, the cytoskeleton-related genes are *Acta1, Tuba1c, Tubb2a,* and *Tubb3*. *Acta1* is a cytoskeletal protein belonging to the actin family, *Tuba1c* is α-tubulin, a part of the cytoskeleton, and *Tubb2a* and *Tubb3* are known as β-tubulin^51,52^. Histone-related genes include *Hist1h2ai, H2afx, H3f3b*^53^. In our previous study, histone acetyltransferase (HAT) inhibition prevented and treated galactose-induced cataracts, suggesting an effect of histone-related genes on cataracts^31,54^. These findings suggest that in the galactose-induced cataract model, oxidative stress induces ER stress, leading to EMT and apoptosis, which, in turn, results in cataracts (Fig. 5). On the other hand, glutamate may have reduced opacity by inhibiting oxidative stress upstream and reversing abnormalities in cell proliferation and the cytoskeleton.

## Discussion

Cataract is an eye disease that results in vision loss or blindness due to opacity of the lens. The current mainstream treatment involves surgery to remove the lens opacity and replace it with an intraocular lens. Cataracts are roughly classified into three types based on the site of opacity: nuclear cataract, posterior subcapsular cataract, and cortical cataract^55^. In the past, drugs such as lanosterol and sterol-related compounds (VP1-001) have been reported to be effective in the treatment of cataracts at the animal experimental level^56,57^. These drugs are thought to have treated nuclear opacity by enhancing the chaperone activity of α-crystallin and reversing protein aggregation. However, the therapeutic effects of these drugs are observed in nuclear cataracts, and their effects on cortical cataracts, as seen in diabetic cataracts, are not clear. In addition, preventive approaches to diabetic cataracts include AR inhibitors, glycation inhibitors, and antioxidants, but none of these drugs has yet been formulated to offer a fundamental treatment^58–60^. Therefore, we investigated whether glutamate, which is expected to have antioxidant effects similar to those of pyruvate, could reduce opacity in a galactose-induced cataract model.

To date, the effect of glutamate on cataracts has not been determined. Subcutaneous administration of monosodium L-glutamate to neonatal mice causes cataracts^29^. Glutamate is also known as an inhibitor of the cystine/glutamate antiporter, and the addition of elastin, a similar inhibitor, to lens epithelial cells induces ferroptosis, which is considered to be a contributing factor in cataracts^61^. On the other hand, glutamate may offer antioxidant effects via various pathways. α-KG is reported to be converted by glutamate through the action of specific enzymes to remove ROS^24^. Glutamate is also a constituent of GSH with antioxidant properties^27^. Furthermore, proline, a metabolite of glutamate, has a preventive effect against cataract induced by H_2_O_2_^28^. In the present study, rat lenses were cultured in galactose medium to induce opacity formation, and then glutamate was added, which reduced the opacity in the lens cortex at all concentrations of 1 mM, 5 mM, 20 mM, and 40 mM (Fig. 1b). Since pyruvate has been reported to prevent diabetic cataracts in the past, a similar pathway could potentially be used to decrease opacity. There is also concern that the addition of glutamate may increase the osmotic pressure of the medium. However, in our previous experiments using glucose medium to examine the effects of osmotic changes on cells, the difference in effects on cells under no osmotic conditions and under hyperosmotic conditions was minimal^62^. Therefore, in this experimental system, we do not believe that the addition of glutamate has any effect on the change in osmotic pressure because the lenses are exposed to high osmotic stress in the Galactose medium at the beginning of the culture stage.

Next, microarray analysis was used to extract genes involved in cataract treatment through a comprehensive analysis of gene expression. Using samples in which the addition of glutamate decreased the opacity, genes whose expression increased due to galactose treatment and decreased with the addition of glutamate were extracted. Quantitative measurement by RT-qPCR was performed on the extracted gene group, and the variation in the expression levels of 41 genes was confirmed (Fig. 3). Furthermore, STRING analysis was used to identify genes with similar biological functions, and they were categorized into 5 major groups (Fig. 4). Genes related to oxidative stress, ER stress, and apoptosis include *Atf3, Ddit3,* and *Txnip*. Depletion of GSH and overproduction of ROS in the lens of major intrinsic protein (MIP) mutant mice result in increased expression of *Atf3* and *Ddit3*^63^. Furthermore, it has been suggested that *Atf3* and *Ddit3* are activated by ER stress and induce apoptosis in UV-exposed LECs^64^. *Txnip* binds to thioredoxin, which has antioxidant activity, and inhibits its activity^43^. *Txnip* is also known to respond to ER stress and induce apoptosis^65^. Oxidative stress induces ER stress and UPR in LECs under high-sugar conditions, resulting in increased ROS formation and apoptosis^44^. Furthermore, increased apoptosis of LECs has been implicated in the development of diabetic cataracts in both human and animal models^66,67^. Next, EMT is a phenomenon in which epithelial cells acquire mesenchymal cell traits, and it has also been suggested to be associated with cellular infiltration and fibrosis^68^. In LECs, EMT is known to be involved in cataracts via AR and is induced by ER stress^69^. Therefore, EMT in the lens may be caused by ER stress induced by oxidative stress. Thus, oxidative stress, ER stress, apoptosis, and EMT were considered important in the pathogenesis of galactose-induced cataracts in this study (Fig. 5).

We have previously reported that the addition of HAT inhibitors and Atm inhibitors reversed the opacity in galactose-induced cataracts^30,31^. Therefore, we compared the genes whose expression level variation was confirmed by RT-qPCR using samples in which opacity was reduced by the addition of HAT inhibitors and Atm inhibitors with those whose expression level variation was confirmed in this study. The number of genes in common with the analysis of any of the HAT inhibitors (C646 + CPTH2, CBP30 + CPTH2, TH1834) was 10 genes (*A3galt2, Acta1, Csrp1, H3f3b, Hebp2, Mki67, Ppdpf, Prtfdc1, Slc16a6, Tuba1c*). The number of genes in common with the analysis of any of the Atm inhibitors (AZD0156, KU55933) was 8 genes (*A3galt2, Acta1, Csrp1, Eif1a, Hspb8, Mki67, Tuba1c, and Tubb3*). In addition, the number of genes in common between the analysis of Glutamate, HAT inhibitors, and Atm inhibitors was 5 genes (*A3galt2, Acta1, Csrp1, Mki67, and Tuba1c*). Among them, *Acta1, Csrp1,* and *Tuba1c* are involved in the cytoskeleton^51,52,70^. Furthermore, *Mki67* is involved in cell proliferation^45^ and a network of genes involved in cell proliferation was also formed in this analysis (Fig. 4). PCNA positive cells were observed in multi-layered epithelium in the galactose diet-loaded model, suggesting that abnormal cell proliferation is involved in cataracts^71^. On the other hand, in lens sections with reduced opacity, the vacuoles are crushed toward the lens nucleus, suggesting that cell proliferation may have contributed to the reduction in opacity^31^. Therefore, moderate cell proliferation is considered important for the treatment of galactose-induced cataracts. In addition to the model in Fig. 5, the above results suggest that the disruption of LECs due to abnormalities in the cytoskeleton and cell proliferation may be a common pathogenic mechanism in galactose-induced cataractogenesis. In addition, it is suggested that the regulation of cytoskeleton and cell proliferation by the addition of glutamate induces cell differentiation of normal LECs and decreases their opacity.

In this study, we found that glutamate is effective in reducing opacity in galactose-induced cataracts. In addition, gene expression analysis revealed genes related to oxidative stress, ER stress, apoptosis, EMT, cell proliferation, cytoskeleton, and histones. Further research targeting these genes may lead to a better understanding of the mechanisms underlying the onset of cataract.

## Materials and methods

### Animals

Six-week-old male Sprague Dawley (SD) rats were purchased from Sankyo Laboratory Service and used for the experiments. All experiments were approved by the Animal Research Committee of the University of Fukui (Approval number: 28091) and conducted in accordance with the University of Fukui regulations on animal experiments and Association for Research in Vision and Ophthalmology Statement for the Use of Animals in Ophthalmic and Vision Research. This study was reported in accordance with the ARRIVE guidelines.

### Ex vivo assays

The rats were euthanized by CO_2_ and then the lens was removed. All lenses were incubated for 3 to 4 days in 2 mL of M199 medium (Sigma-Aldrich) containing 0.1% BSA and 30 mM Galactose using an incubator set at 5% CO_2_ and 37 °C to induce opacity, as previously reported^30^. After opacity was induced, images were taken under a microscope and the same medium was replaced. To one medium, 16 µL of Sodium Hydrogen L (+)-Glutamate monohydrate (Wako) dissolved in sterile water was added at final concentrations of 1, 5, 20, and 40 mM, and to the other medium, only 16 µL of sterile water was added. The lenses were further incubated for 2–3 days and photographed under a microscope. Control samples that did not induce cataracts were incubated with sterile water instead of galactose for 4 or 6 days.

### Microscopic observation

Photographs of the lens were taken in the darkroom using an SZX12 stereomicroscope with a DP58 camera (Olympus) attached, as previously shown^54^. Photographs were taken in 35 mm petri dishes containing 7 mL of PBS. A weighted average was calculated from the brightness (0–255) of the cortex area of the lens that was opacified by incubation with galactose, and the weighted average was again calculated from the brightness of the same area in the lens after addition of the inhibitor and further incubation^30^. The change in opacity was calculated by subtracting the value after the addition of the inhibitor from the value before the addition of the inhibitor.

### Microarray data analysis

Microarray analysis was performed using control samples cultured for 4 or 6 days (n = 2), cataract samples cultured in galactose medium for 6 days (n = 3) and treated samples with glutamate addition (n = 2). A GeneChip Rat Gene 2.0 ST array chip (Thermo Fisher Scientific) was used to perform microarray experiments as described previously^72^. First, data from all samples were normalized with the Robust Multi-array Average algorithm to exclude probes that did not correspond to genes. Next, genes with signal values less than 5 in all samples were excluded. Signal values for each condition were normalized by the mean value between each sample. Genes whose expression increased more than twofold from the Control means to the Galactose means and whose expression decreased more than 2.25-fold from the Galactose means to the Glutamate means were selected as important genes. The extracted genes were subjected to biological functional analysis using STRING (https://string-db.org/).

### RNA extraction, cDNA preparation, and real-time RT-qPCR

Lens RNA extraction and real-time RT-qPCR were conducted using the same methods as described previously^73^. The primers used are listed in (Supplementary Table S2). Gene expression levels were normalized against *Gapdh* expression levels. To assess the difference between the galactose samples and the control and glutamate samples, genes whose expression levels decreased by more than 10% from Galactose to Glutamate, with the increased expression of the genes from Control to Galactose set as 100%, were considered important genes for the reduction in opacity.

### Supplementary Information

Supplementary Information 1.

Supplementary Information 2.

## Data Availability

Microarray data are available in the GEO repository under the accession number GSE227475 (https://www.ncbi.nlm.nih.gov/geo/query/acc.cgi?acc=GSE227475).
