# Supplementary material for: Glutamate is effective in decreasing opacity formed in galactose-induced cataract model
Source: Sci Rep. 2024 Feb 19;14:4123. doi: 10.1038/s41598-024-54559-y (PMC10876653; doi:10.1038/s41598-024-54559-y)
Supplement: Supplementary file 1 — Supplementary Information 1. [file 41598_2024_54559_MOESM1_ESM.docx]

Glutamate is effective in decreasing opacity formed in galactose-induced cataract model

Masaru Takashima^1^, Shunki Yamamura^1^, Chie Tamiya^1^, Mayumi Inami^2^, Yoshihiro Takamura^3^, Masaru Inatani^3^ and Masaya Oki^1,4 *^

^1^Department of Industrial Creation Engineering, Graduate School of Engineering, University of Fukui, Fukui, Japan;

^2^Technical Division, School of Engineering, University of Fukui, Fukui, Japan

^3^Department of Ophthalmology, Faculty of Medical Sciences, University of Fukui, Fukui, Japan;

^4^Life Science Innovation Center, University of Fukui, Fukui, Japan

Correspondence and requests for materials should be addressed to M.O.(email: ma4sa6ya@u-fukui.ac.jp)

**
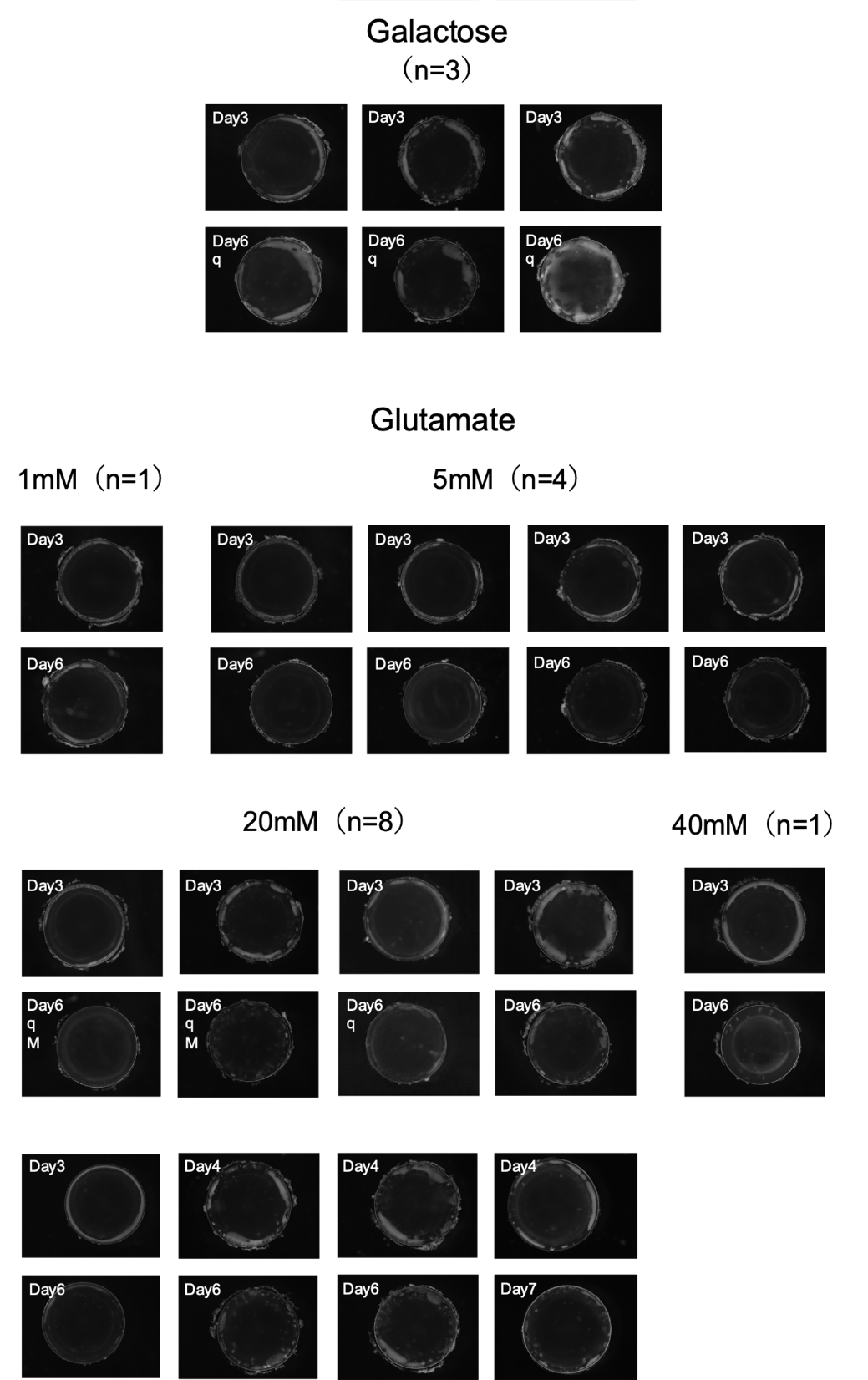
**

**Supplementary Figure S1. Lens photographs used for opacity quantification.**

Photographs of lenses used for opacity quantification after addition of various concentrations of glutamate. The upper panel shows lens photographs before and the lower panel shows lens photographs after addition of the inhibitor." q" indicate samples used for RT-qPCR and "M" indicate samples used for microarray analysis.

**Supplementary Table S1. List of 51 genes for which RT-qPCR was performed**

| **Gene** | **Description** |
| --- | --- |
| *A3galt2* | alpha 1,3-galactosyltransferase 2 |
| *Acta1* | actin, alpha 1 |
| *Ankrd1* | ankyrin repeat domain 1 |
| *Atf3* | activating transcription factor 3 |
| *Csrp1* | cysteine and glycine-rich protein 1 |
| *Ctsl* | cathepsin L |
| *Ddit3* | DNA-damage inducible transcript 3 |
| *Eif1a* | eukaryotic translation initiation factor 1A |
| *Gbp5* | guanylate binding protein 5 |
| *H2afx* | H2A histone family, member X |
| *H3f3b* | H3 histone, family 3B |
| *Hebp2* | heme binding protein 2 |
| *Hist1h2ai* | histone cluster 1, H2ai |
| *Hist1h4m* | histone cluster 1, H4m |
| *Hn1* | hematological and neurological expressed 1 |
| *Hspb8* | heat shock protein B8 |
| *Ifi27* | interferon, alpha-inducible protein 27 |
| *Imp3* | U3 small nucleolar ribonucleoprotein |
| *Lcn2* | lipocalin 2 |
| *Mki67* | marker of proliferation Ki-67 |
| *Mt1a* | metallothionein 1a |
| *Mt1m* | metallothionein 1M |
| *Mt2A* | metallothionein 2A |
| *Nhp2l1* | NHP2-like protein 1 |
| *Nme2* | NME/NM23 nucleoside diphosphate kinase 2 |
| *Nr4a1* | nuclear receptor subfamily 4, group A, member 1 |
| *Pcna* | proliferating cell nuclear antigen |
| *Pdpn* | podoplanin |
| *Pmf1* | polyamine-modulated factor 1 |
| *Ppdpf* | pancreatic progenitor cell differentiation and proliferation factor |
| *Prr13* | proline rich 13 |
| *Prtfdc1* | phosphoribosyl transferase domain containing 1 |
| *Rcan1* | regulator of calcineurin 1 |
| *S100a1* | S100 calcium binding protein A1 |
| *Sdcbp* | syndecan binding protein |
| *Sesn2* | sestrin 2 |
| *Siva1* | apoptosis-inducing factor |
| *Slc16a14* | solute carrier family 16, member 14 |
| *Slc16a6* | solute carrier family 16, member 6 |
| *Slc20a1* | solute carrier family 20, member 1 |
| *Stat3* | signal transducer and activator of transcription 3 |
| *Timp1* | TIMP metallopeptidase inhibitor 1 |
| *Tmco4* | transmembrane and coiled-coil domains 4 |
| *Tmem140* | transmembrane protein 140 |
| *Tuba1c* | tubulin, alpha 1C |
| *Tubb2a* | tubulin, beta 2A class IIa |
| *Tubb3* | tubulin, beta 3 class III |
| *Txnip* | thioredoxin interacting protein |
| *Tyms* | thymidylate synthetase |
| *Vamp3* | vesicle-associated membrane protein 3 |
| *Zfp36* | zinc finger protein 36 |

**Supplementary Table S2. List of Primer used for real-time RT-qPCR.**

| Gene | Forward Primer | Reverse Primer |
| --- | --- | --- |
| *Gapdh* | 5'-GAGACAGCCGCATCTTCTTGT-3' | 5'-CGACCTTCACCATCTTGTCTATGA-3' |
| *A3galt2* | 5'-GGGACTCAGGGCCAAGAAG-3' | 5'-CAGGCCTAGGAGACCAAACG-3' |
| *Acta1* | 5'-TCAGGCGGTGCTGTCTCTCT-3' | 5'-TCCCCAGAATCCAACACGAT-3' |
| *Ankrd1* | 5'-CGGCTCTTGATGACCTTCG-3' | 5'-GCATTCTCCTTGAGGCTGTC-3' |
| *Atf3* | 5'-CACCTTTGCCATCGGATGTCC-3' | 5'-CTTTCCCGCCGCCTCCTT-3' |
| *Csrp1* | 5'-CCAGTGCGAGGGCAACA-3' | 5'-TGCAAACCATGCACAGGAA-3' |
| *Ctsl* | 5'-TGGAGAGAAAAGGGTTGTGTGA-3' | 5'-AGCCCAGCAAGAACCACACT-3' |
| *Ddit3* | 5'-TCATCCCCAGGAAACGAAGA-3' | 5'-AGCTAGGGATGCAGGGTCAA-3' |
| *Eif1a* | 5'-GACCACCGTAGGCTGGAGTAAG-3' | 5'-CGGACAATTAGCGTCTAAAGCA-3' |
| *Gbp5* | 5'-CGCACAGGCAAATCCTACCT-3' | 5'-GCCCACGGAAAAGCCTTT-3' |
| *H2afx* | 5'-CAGTTGACCCTGTCGTCATTTG-3' | 5'-TGGAAAAATTGGCTTCACGTT-3' |
| *H3f3b* | 5'-AGGGCCTCAGACTTCAGCTTT-3' | 5'-TCCTAGCGGTCTGCTTGGTT-3' |
| *Hebp2* | 5'-CCGCCTTGTGGCTCAAAC-3' | 5'-ACGCACGGCCCTGAAA-3' |
| *Hist1h2ai* | 5'-TGCATTTCTGTGGACACATTACC-3' | 5'-AGTGGAATAATTTGTCGTAGGAAGTACA-3' |
| *Hist1h4m* | 5'-CAAAGGCGGCGCTAAGC-3' | 5'-TAGTGATGCCCTGGATGTTGTC-3' |
| *Hn1* | 5'-CAAAGTGGCCATACCTCACATC-3' | 5'-CAGTGGCCATTGGCTTCCT-3' |
| *Hspb8* | 5'-TCTCCAGAGGGTCTGCTCAT-3' | 5'-GCAGGTGACTTCCTGGTTGT-3' |
| *Ifi27* | 5'-TGCACTTGGATCTGCATTGG-3' | 5'-GGCAGGGAGGCTGGATAAAA-3' |
| *Imp3* | 5'-ACCGATCCCGCCTTTCTC-3' | 5'-TCCACCCAGGTGACAAAGTCT-3' |
| *Lcn2* | 5'-GCAGTGGCCTGATGGTTCA-3' | 5'-TCTGGCAACAGGAAAGATGGA-3' |
| *Mki67* | 5'-ATTTCAGTTCCGCCAATCC-3' | 5'-GGCTTCCGTCTTCATACCTAAA-3' |
| *Mt1a* | 5'-CAGCAGCCTGACTGCCTTCT-3' | 5'-CATTCCGAGATCTGGTGAATCTG-3' |
| *Mt1m* | 5'-GCTCCTAGAACTCTACAGCGATCTC-3' | 5'-GGGTCCATGGCGAATGG-3' |
| *Mt2A* | 5'-GCTCCTAGAACTCTACAGCGATCTC-3' | 5'-GGGTCCATGGCGAATGG-3' |
| *Nhp2l1* | 5'-ATGACGGAGGCTGATGTGAAC-3' | 5'-TTGGTGAGGTGGGCATCTG-3' |
| *Nme2* | 5'-GAAGCCAGGCACCATTCGT-3' | 5'-ACTGCCGTGAATGATGTTCCT-3' |
| *Nr4a1* | 5'-GCGGCTTTGGTGACTGGAT-3' | 5'-GGCCATGTCGATCAGTGATGAG-3' |
| *Pcna* | 5'-CTCACGTCTCCTTAGTGCAGCTT-3' | 5'-CGATCGCAGCGGTATGTGT-3' |
| *Pdpn* | 5'-CCACGGACAAGAAAACAACTCA-3' | 5'-TCTTATCTGTGGTCTGCGTTTCA-3' |
| *Pmf1* | 5'-AGTCGCGGGCAAGAGCTA-3' | 5'-GGGTTCAACTGGTGCAAGTGT-3' |
| *Ppdpf* | 5'-GGAAATCCACCCTCCCATTC-3' | 5'-TGCCGAGTGCTCAGGAGACT-3' |
| *Prr13* | 5'-CCCTGTCCTCCCGGAATT-3' | 5'-TCGACACGGAGGGAAAGC-3' |
| *Prtfdc1* | 5'-CAGCAGCATTGAGAAATACAAACC-3' | 5'-CGCTGTCCTCTTCACCAATAAA-3' |
| *Rcan1* | 5'-CGCGTTCTGATTCCCACAT-3' | 5'-TGACCAGCCACTTGCACAGT-3' |
| *S100a1* | 5'-CATCCCACCTGTATCTCCCTATG-3' | 5'-CGCCTTTGGTGCATGTTG-3' |
| *Sdcbp* | 5'-AAACGGCCTTCTCACTGATCA-3' | 5'-GAGCATCCTTCAAGCCAATGA-3' |
| *Sesn2* | 5'-TTTCGTGCCCAGGATTATACCT-3' | 5'-GGGTAGAGCCGCTGGATCA-3' |
| *Siva1* | 5'-TGTTCGCCGAGCGCTACT-3' | 5'-TGGAAAAGGAGCTGCTTGGT-3' |
| *Slc16a14* | 5'-TGCTGAAGTACCTGTGTGCAGAAT-3' | 5'-CAGAGCACCTTGGATGAACATG-3' |
| *Slc16a6* | 5'-CCGCGCTGCATTCTTACTGT-3' | 5'-CCGATCCTCCCGAACACTT-3' |
| *Slc20a1* | 5'-CCGTCAGCAACCAGATCAACTC-3' | 5'-CCCATGCAGTCTCCCACCTTG-3' |
| *Stat3* | 5'-AGCTCTTAGGGCCTGGTGTGAACTACT-3' | 5'-GGATGGCCCTCTCCCGCTCCTTGCTGA-3' |
| *Timp1* | 5'-CGCAGCGAGGAGTTTCTCAT-3' | 5'-GGCAGTGATGTGCAAATTTCC-3' |
| *Tmco4* | 5'-CCACCGGTCTGCTGAGGTT-3' | 5'-CCCTGTTGCCTCGAGAGAAG-3' |
| *Tmem140* | 5'-AAGAACCACTGGGAGAACTGCTA-3' | 5'-TGAACCCTTGCCATCCCATA-3' |
| *Tuba1c* | 5'-TTTCGCGGACCACTTCAAG-3' | 5'-TGGCCAACGTGGATGGA-3' |
| *Tubb2a* | 5'-GCCAATGCGGCAACCA-3' | 5'-GCCATGCTCATCGCTTATCA-3' |
| *Tubb3* | 5'-GGGCCTTTGGACACCTATTCA-3' | 5'-CCCTTTGGCCCAGTTGTTG-3' |
| *Txnip* | 5'-TGACCGTGCAGCCTGTGA-3' | 5'-CGCTCCCACGGTTAGTCAAC-3' |
| *Tyms* | 5'-TGTGCCTGGAACCCAAAAG-3' | 5'-CAGAGGGCATGGCAAGGA-3' |
| *Vamp3* | 5'-GCGCCTCGCAGTTTGAAA-3' | 5'-CTTGCAGTTCTTCCACCAATACTTT-3' |
| *Zfp36* | 5'-TCGCGCCACCATGGAT-3' | 5'-GTCATGGCTCATCGACATAAGG-3' |

**Supplementary Dataset S1. List of 265 genes that were increased more than 2-fold from Control to Galactose and decreased more than 1.5-fold from Galactose to Glutamate.**

The ten columns on the right of the gene name display the signal value (log2) in each sample. Average indicates the mean value of replicate samples. The column for Control vs Galactose indicates the fold increase in expression from Control to Galactose. The column of Galactose vs Glutamate shows the fold decrease in expression from Galactose to Glutamate.
